# Congenital goitrous hypothyroidism is caused by dysfunction of the iodide transporter SLC26A7

**DOI:** 10.1038/s42003-019-0503-6

**Published:** 2019-07-24

**Authors:** Jun Ishii, Atsushi Suzuki, Toru Kimura, Michihiro Tateyama, Tatsushi Tanaka, Takuya Yazawa, Yu Arimasu, I-Shan Chen, Kohei Aoyama, Yoshihiro Kubo, Shinji Saitoh, Haruo Mizuno, Hiroshi Kamma

**Affiliations:** 1 Department of Pathology, Kyorin University School of Medicine, Tokyo, Japan; 2 Department of Pathology, Dokkyo Medical University, Tochigi, Japan; 3 Department of Pediatrics and Neonatology, Nagoya City University Graduate School of Medical Sciences, Nagoya, Japan; 4 Department of Pharmacology and Toxicology, Kyorin University School of Medicine, Tokyo, Japan; 5 Division of Biophysics and Neurobiology, Department of Molecular and Cellular Physiology, National Institute for Physiological Sciences, Okazaki, Japan; 6 Department of Pediatrics, International University of Health and Welfare, School of Medicine, Narita, Japan

**Keywords:** Cell biology, Genetics, Endocrinology, Pathogenesis, Cell biology

## Abstract

Iodide transport and storage in the thyroid follicles is crucial for thyroid hormone synthesis. Pendrin, the iodide exporter that transports iodide to thyroid follicles, is responsible for Pendred syndrome, a disorder characterized by congenital hypothyroidism and hearing loss. However, thyroid hormone levels are basically normal in patients with Pendred syndrome, indicating the presence of another unknown iodide transporter. Here, we show that SLC26A7 is a novel iodide transporter in the thyroid. We observe that SLC26A7 is specifically expressed in normal thyroid tissues and demonstrate its function in iodide transport. Using whole-exome sequencing, we also find a homozygous nonsense mutation in *SLC26A7* (c.1498 C > T; p.Gln500Ter) in two siblings with congenital goitrous hypothyroidism. The mutated SLC26A7 protein shows an abnormal cytoplasmic localisation and lacks the iodide transport function. These results reveal that SLC26A7 functions as a novel iodide transporter in the thyroid and its dysfunction affects thyroid hormonogenesis in humans and causes congenital goitrous hypothyroidism.

## Abstract (web summary)

Jun Ishii and Atsushi Suzuki et al. report the identification of SLC26A7 as a novel iodide transporter expressed in the thyroid. They identify a nonsense mutation in *SLC26A7* in siblings with congenital hypothyroidism and goitre using whole-exome sequencing, implicating this transporter in disease.

## Introduction

Thyroid hormone is involved in metabolism^1^ and is an essential hormone for the proper functioning of the organism. Moreover, the iodide transporter plays an important role in the synthesis of thyroid hormone. Thyroid hormone is synthesised in the lumen of the thyroid follicle from 3 to 4 iodides and two condensed tyrosine residues. After its uptake via the diet, iodide in the circulating blood is transported by the iodide transporter via thyroid follicular cells to the thyroid lumen. Two thyroid iodide transporters are widely known: the iodide importer, also known as the sodium/iodide symporter (NIS, SLC5A5), which transports iodide from circulating blood to follicular cells; and the iodide exporter, also known as pendrin (SLC26A4), which transports iodide from the follicular cells to the lumen^2^. However, another luminal iodide transporter may exist. Although patients with Pendred syndrome carry biallelic loss-of-function mutations in the pendrin gene *SLC26A4* and are characterised by congenital hypothyroidism and hearing loss, they can maintain normal thyroid hormone levels, as can *Slc26a4* knockout mice^3,4^. Thus, it is assumed that mammals have another iodide transporter, aside from SLC26A4^5,6^.

Based on the analysis of gene expression databases (GeneMANIA; http://genemania.org/, IST Online; http://ist.medisapiens.com/), we found that the *SLC26A7* gene is usually co-expressed with thyroid-specific genes, including thyroglobulin and thyroid peroxidase, thyroid transcription factor 1, thyroid stimulating hormone receptor, paired box 8 and forkhead box E1. We also found that SLC26A7 expression was specific to the kidney and thyroid gland. In addition, *Slc26a7* knockout mice displayed hypothyroidism with histological observations of hyperplastic thyrotrophs^7,8^. Although SLC26A7 has been described as a Cl^−^/HCO_3_^−^ exchanger in the kidney^9^ or Cl^−^ channel^10^, transporting molecules important for thyroid hormone synthesis^8^, no comprehensive studies have addressed whether SLC26A7 represents a novel iodide transporter in the thyroid.

In this study, we show that SLC26A7 is indeed a novel iodide transporter in the thyroid. Moreover, we identified a homozygous nonsense mutation in *SLC26A7* (c.1498 C > T; p.Gln500Ter) in two siblings with congenital goitrous hypothyroidism. The mutation impaired the localisation of SLC26A7 to the cell membrane and abolished its iodide transport activity. These results collectively support the notion that SLC26A7 is another iodide transporter in the thyroid together with SLC26A4, and that its dysfunction affects thyroid hormonogenesis in humans and causes congenital goitrous hypothyroidism.

## Results

### SLC26A7 is expressed in thyroid tissues

**Fig. 1:**
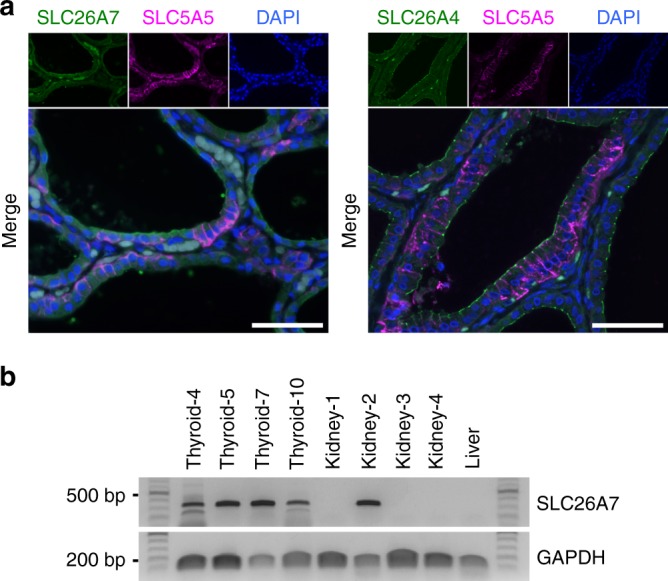
Expression of SLC26A7 in thyroid tissues. **a** Immunofluorescence staining of SLC26A7 (green) and SLC5A5 (magenta), or SLC26A4 (green) and SLC5A5 (magenta) in human thyroid tissue. 4ʹ,6-diamidino-2-phenylindole, DAPI (blue) marks cell nuclei. Scale bar, 50 μm. **b** SLC26A7 messenger RNA expression in thyroid, kidney, and liver tissues. Glyceraldehyde 3-phosphate dehydrogenase (GAPDH) served as an internal control

We first conducted an immunohistochemical analysis to confirm SLC26A7 expression in human thyroid tissues. SLC26A7 signals were detected predominantly on the apical side facing the follicular lumen in thyroid follicular cells (Fig. 1a, Supplementary Fig. 1). Unlike with SLC5A5, the staining pattern of SLC26A7 resembled that of SLC26A4. To verify the validity of our immunohistochemical results, reverse transcription polymerase chain reaction (RT-PCR) analysis was performed to confirm SLC26A7 mRNA expression in the thyroid tissues. All analysed thyroid samples indicated SLC26A7 expression (Fig. 1b), while only one kidney sample showed SLC26A7 expression. In addition, one liver tissue sample did not display SLC26A7 expression. The uncropped gel images are shown in Supplementary Fig. 2. A summary of the immunohistochemical and RT-PCR analysis is shown in Supplementary Table 1. These results clearly indicate that thyroid tissues express SLC26A7.

### SLC26A7 is present on the apical and lateral sides but not on the basal side

**Fig. 2:**
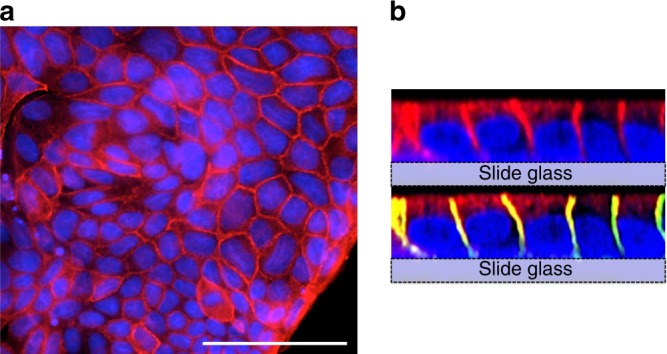
Localisation of SLC26A7. **a** Immunofluorescence analysis of FLAG (red) in FLAG-tagged SLC26A7-transfected MDCK cells. 4ʹ,6-diamidino-2-phenylindole, DAPI (blue) marks cell nuclei. Scale bar, 50 μm. **b** Confocal image of FLAG-tagged SLC26A7-transfected MDCK cells. The top image was stained with FLAG (red) and DAPI (blue). The lower image was stained with FLAG and DAPI, sodium potassium ATPase (green)

To analyse the subcellular localisation and the iodide transport function of SLC26A7, SLC26A7 or SLC26A4 complementary DNA (cDNA) was transfected with or without SLC5A5 into MDCK and Nthy, COS-7, WRO cells using a tetracycline induction system and referred to as MDCK-SLC26A7, MDCK-SLC26A4, MDCK- SLC5A5/SLC26A7 and MDCK- SLC5A5/SLC26A4. Since the original cells used in this study merely uptake iodide, the iodide importer *SLC5A5* was transfected into the cells together with *SLC26A7* or *SLC26A4*. *SLC5A5* alone was also transfected into these cells as a control. The expression of the transgene was confirmed by western blot analysis and the representative results from MDCK cells are shown in Supplementary Fig. 3. SLC26A7, SLC26A4 or SLC5A5 proteins were strongly detected following each transfection. Although SLC5A5 expression was slightly decreased by the co-expression of the SLC26A7 or SLC26A4 transgenes in MDCK and Nthy cells, this phenomenon was not evident in COS-7 and WRO cells (Supplementary Fig. 4). In addition, reduced SLC5A5 mRNA expression following SLC26A7 induction was confirmed in MDCK, but not Nthy cells (Supplementary Fig. 5); thus, we speculated that the downregulation of SLC5A5 following SLC26A7 induction is not a direct effect of SLC26A7. Because SLC5A5 expression remained high in cells transfected with *SLC26A7* (MDCK-SLC5A5/SLC26A7) compared with those transfected with an empty vector (MDCK-Empty), the following experiments were performed using these transfectants. At first, the cellular localisation of SLC26A7 was analysed by fluorescence immunocytochemistry. FLAG-conjugated SLC26A7 was strongly detected at the cell membrane (Fig. 2a, Supplementary Fig. 6, Supplementary Fig. 7); moreover, underlining epithelial polarity, SLC26A7 was detected on the apical and lateral side, whereas staining of the basal side was not confirmed (Fig. 2b).

### SLC26A7 functions as an iodide transporter

**Fig. 3:**
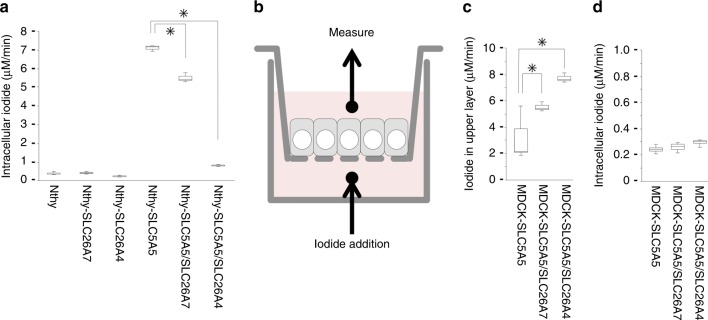
Radio iodide transport and uptake assays. **a** Measured intracellular iodide after supplementation with radioactive NaI for 5 min. SLC26A7 or SLC26A4 was induced by doxycycline 24 h before the assay. Data represent the means ± SD. *n* = 3 biologically independent samples. *p < 0.05. **b** A schematic diagram of the iodide transport assay using the bicameral system. **c**, **d** Measured iodide in the top chamber (**c**) and inside the cell (**d**) after supplementation with radioactive NaI for 45 min. SLC26A7 or SLC26A4 was induced by doxycycline 24 h before the assay. Data indicate the means ± SD. *n* = 4 biologically independent samples. *P < 0.05

Next, we analysed the iodide transport function of SLC26A7 in the Nthy thyroid follicular cell line. We considered Nthy cells to be optimal for use in this experiment, rather than MDCK cells, owing to the greater intracellular iodide accumulation in Nthy cells. Radioactive cellular NaI was measured 5 min after its addition to the culture medium (Fig. 3a). The iodide import capacity of Nthy cells was not very high, and a single transfection with either the *SLC26A7* or *SLC26A4* gene did not affect iodide uptake. On the contrary, transfection with the sodium-iodide symporter *SLC5A5* greatly enhanced iodide uptake; however, the intracellular iodide level was significantly lower in the *SLC5A5/SLC26A7* or *SLC5A5/SLC26A4* double transfectant than that in the *SLC5A5* single transfectant (*SLC5A5/SLC26A7* double transfectant against *SLC5A5* single transfectant: *P* = 0.0112, Student’s *t* test; *SLC5A5/SLC26A4* double transfectant against *SLC5A5* single transfectant: *P* = 0.0004, Student’s *t* test). Although there was not a significant difference, similar tendencies were also seen in the study by Cangul et al. using HEK293 cells^8^. The release of iodide from of the cell by SLC26A4 was greater than that from SLC26A7; however, these data suggest that SLC26A7 exports intracellular iodide to the outside of the cell and does not contribute to the uptake of iodide into the cell. Iodide efflux by SLC26A7 was also confirmed via the Sandell–Kolthoff reaction in Nthy and COS-7 cells (Supplementary Table 2).

Next, we examined the iodide transport function of SLC26A7 by using a bicameral culture system^11^ (Fig. 3b). In this system, the cells were grown on semipermeable cell culture insert filters and the culture medium was divided into two chambers across the cell sheets. Then, the radioactive NaI moving from the bottom to the top chamber through the cell sheets was measured. To conduct this analysis, we used MDCK cells that form tight junctions and display sheet-like growth patterns. Other cell lines used in this study did not present these characteristics. After confirming the formation of tight junctions by measuring the transepithelial electrical resistance, radioactive NaI was added to the bottom chamber, and the amount of iodide transported to the top chamber was measured after 45 min (Fig. 3c). Compared with the *SLC5A5* single transfectant, the *SLC5A5/SLC26A7* or *SLC5A5/SLC26A4* double transfectant transported more iodide to the top chamber (*SLC5A5/SLC26A7* double transfectant against *SLC5A5* single transfectant: *P* = 0.0255, Student’s *t* test; *SLC5A5/SLC26A4* double transfectant against *SLC5A5* single transfectant: *P* = 0.0005, Student’s *t* test). The intracellular iodide levels were similar for all transfectants (Fig. 3d). Although the amount of transported iodide in the *SLC5A5/SLC26A7* transfectant was less than that in the *SLC5A5/SLC26A4* double transfectant, these results indicate that SLC26A7 can efflux iodide from the inside to the outside of the cell through the apical side of the cell.

### A homozygous nonsense mutation in *SLC26A7* causes congenital hypothyroidism

**Fig. 4:**
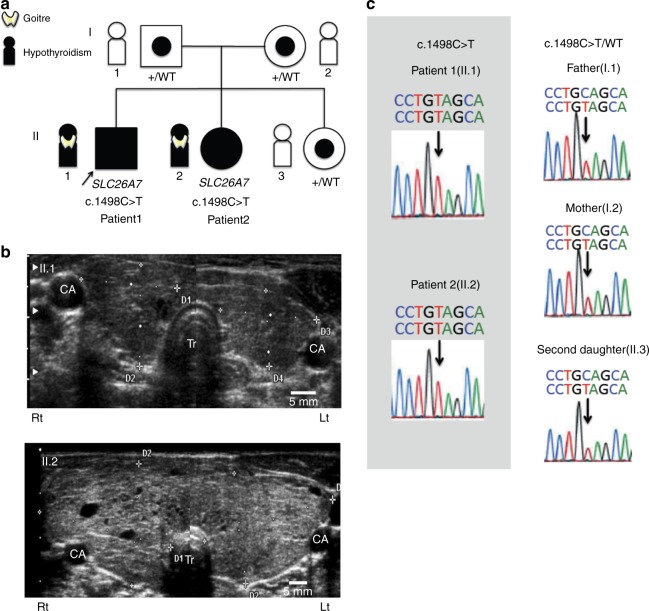
Homozygous mutations in *SLC26A7* cause goitrous hypothyroidism. **a** Family pedigree. Affected patients carry a homozygous nonsense mutation in the *SLC26A7* gene. All other unaffected family members have a heterozygous mutation. Squares, males; circles, females; filled symbols, affected individuals; target symbols, carrier; arrow, the proband. **b** Thyroid ultrasonography of affected patients. The upper photo (II.1) represents patient 1 at 7 years of age when the LT4 treatment was resumed. The thyroid gland is slightly enlarged with a right lobe width of 19.2 mm (7.5–15.7) and a thickness of 16.5 mm (6.1–12.9), and a left lobe width of 19.2 mm (7.9–15.1) and thickness of 12.4 mm (5.3–11.4). The reference range for the corresponding age is shown in parentheses. The lower photo (II.2) represents patient 2 at 5 years of age when the diagnosis was made. The thyroid gland is markedly enlarged with heterogeneous inner echo signals defining adenomatous goitre. The right lobe width is 38.9 mm (6.9–13.7) and the thickness is 27.7 mm (5.8–12.0), while the left lobe width is 37.1 mm (7.0–13.4) and the thickness is 22.4 mm (5.0–11.1). CA, common carotid artery; Tr, Trachea; Rt, right; Lt, left. **c** Sanger sequencing results of *SLC26A7* in family members. Note that affected patients show a homozygous C > T substitution (c.1498 C > T) while other family members show a heterozygous substitution

We conducted whole-exome sequencing for two siblings with congenital hypothyroidism and goitre, their healthy sibling, and parents (Fig. 4a). Patient 1, a 15-day-old male neonate, born to unrelated non-consanguineous parents, was referred to the previous institution suspected of congenital hypothyroidism following neonatal mass screening. The clinical diagnosis was hypothyroidism with obvious goitre. The initial laboratory results showed extremely high serum thyroid-stimulating hormone level (TSH > 100 μIU/mL) and low free thyroxine level (FT4, 0.64 ng/dL) (Table 1). Thyroglobulin (Tg) was not tested during the initial examination. At the initiation of therapy, the diameter of the thyroid at its maximal width was 53 mm (reference range: 22–28 mm)^10^ according to thyroid ultrasonography. The severity of his hypothyroidism was moderate as defined by European Society for Paediatric Endocrinology (ESPE) criteria. Levothyroxine (LT4) supplementation for hypothyroidism was initiated at 50 μg/day. However, the necessary dose needed to maintain normal TSH level was gradually decreased. As a result, LT4 supplementation was discontinued at 2 years, and the patient was declared euthyroid until 7 years of age. At 7 years, when he first visited our institution, the laboratory results showed high-serum TSH level (35.8 μIU/mL), an extremely low-serum FT4 level (0.38 ng/dL) and a high-serum Tg level (314 ng/mL). There was palpable goitre (WHO grade 1). Using thyroid ultrasonography, we observed that the thyroid gland was slightly enlarged (Fig. 4b). We, therefore, resumed LT4 supplementation at 30 μg/day. Currently, the patient continues to undergo LT4 supplementation and displays no complications, such as short stature, developmental disabilities or hearing loss.

**Table 1:** Patient characteristics

	Patient 1	Patient 2	Reference values
Age at diagnosis	15 days old	5 years old	
Sex	Male	Female	
Chief complaint for diagnosis	Neonatal screening	Goitre	
*Initial evaluation*			
Serum TSH (µIU/mL)	>100	31.79	0.340–4.220
Serum FT4 (ng/dL)	0.64	0.18	0.77–1.59
Serum FT3 (pg/mL)	4.8	4.72	2.24–3.94
Serum Tg (ng/mL)	N.A.	2600	<54.6
Thyroid morphology	Goitre	Adenomatous goitre	
Developmental disability	No	No	
Adult height (cm)	173.7	161.1	
Initial dose of thyroxine (µg/day)	50	50	
Present dose of thyroxine (µg/day)	100	35	

*TSH,* thyroid stimulating hormone; *FT4,* free thyroxine; *FT3,* free triiodothyronine; *Tg,* thyroglobulin; *N.A.,* not available

Patient 2, the younger sister of the proband, was a 5-year-old girl. She visited our institution due to awareness of goitre. Her neonatal mass screening was normal. The initial laboratory examination showed elevated serum TSH (31.79 μIU/mL), considerably low serum FT4 (0.18 ng/dL), and extremely elevated serum Tg levels (2600 ng/mL) (Table 1). Her hypothyroidism was severe as defined by ESPE criteria. The physical examination revealed a well-nourished girl with a height of 113.4 cm (+0.79 SD) and a weight of 23.15 kg (+1.69 SD). There was a visible and significantly enlarged goitre (WHO grade 2). Using thyroid ultrasonography, we observed that the thyroid gland was markedly enlarged (Fig. 4b). LT4 supplementation was initiated at 50 μg/day. Currently, she continues to take LT4 supplementation and exhibits no complications, the same as the proband.

In the *SLC26A7* gene (NM_001282356.1), we identified a homozygous nonsense mutation (c.1498 C > T; p,Gln500Ter) in patients 1 and 2. This mutation is registered as rs774517670, for which the minor allele frequency is 0.0001173 (East Asia) in the Exome Aggregation Consortium (ExAC) and 0.0008 in the Human Genetic Variation Database (HGVD). The unaffected mother, father, and a sibling carried the mutation in a heterozygous state (Fig. 4c). Furthermore, we identified candidate homozygous or compound heterozygous rare variants in five genes (Table 2, Supplementary Fig. 8), but no de novo or X-linked candidates. Variants other than that in *SLC26A7* were not assessed as pathogenic based on the allele frequency, prediction results and estimated function of the genes. The mutation in our patients was defined as likely pathogenic based on the ACMG guideline.

**Table 2:** Rare variants identified from whole exome sequencing

	Gene	Chr	Region	RefSeq	Mutation							
					Nucleotide	Amino acid	Inheritance^a^	HGMD2017^b^	Reported phenotype	SNP	MAF	SIFT^c^/ PolyPhen2^d^/ Mutation Taster^e^
Homozygous recessive	SLC26A7	8	92378817	NM_001282356.1	c.1498C > T	p.Gln500Ter	P	ND	Anion transporter	rs774517670	0.0001173	−/−/D
							M					
Compound heterozygous	NEB	2	152420386	NM_001164508	c.18530G > A	p.Arg6177His	M	ND	Nemaline myopathy	rs147159176	0.01702	D/P/P
			152520260		c.5565C > A	p.Asp1855Glu	M	ND		rs200468391	0.002325	T/B/P
			152347020		c.25163G > A	p.Arg8388His	P	ND		rs139333406	0.001589	D/P/D
			152512681		c.6481G > A	p.Arg2161Cys	P	ND		rs201758329	0.0001167	T/P/P
	ZNF655	7	99171018	NM_001083956	c.1392G > A	p.Met464IIe	M	ND	Zinc finger protein	ND	0.0004623	B/T/P
			99170144		c.518T > C	p.Phe173Ser	P	ND		rs149822831	0.0156	D/P/D
	FOCAD	9	20990349	NM_017794	c.5232G > A	N/A	M	ND	Adenomatous polyposis coli	rs80118002	0.002143	T/B/D
			20923667		c.2861C > T	p.Pro954Leu	P	ND		rs200166806	0.006023	D/P/D
	GOLGA8K	15	32685249	ENST00000512626	c.1711C > T	p.His571Tyr	M	ND	ND	ND	ND	D/P/D
			32688635		c.984G > C	p.Lys328Asn	P	ND		rs200425540	ND	T/B/P

^a^Inheritance is described as M, Maternal; P, Paternal; M−, mother does not carry

^b^HGMD 2017 are described as DM, disease causing mutation

^c^SIFT are described as D, deleterious; T, tolerated

^d^PolyPhen-2 are described as D, probably damaging; P, possibly damaging; B, benign

^e^Mutation Taster are described as D, disease causing; P, polymorphism. Chr, chromosome. MAF, from ExAC (East Asia). N/A, not applicable (no amino acid change)

### Mutated SLC26A7 shows abnormal localisation

**Fig. 5:**
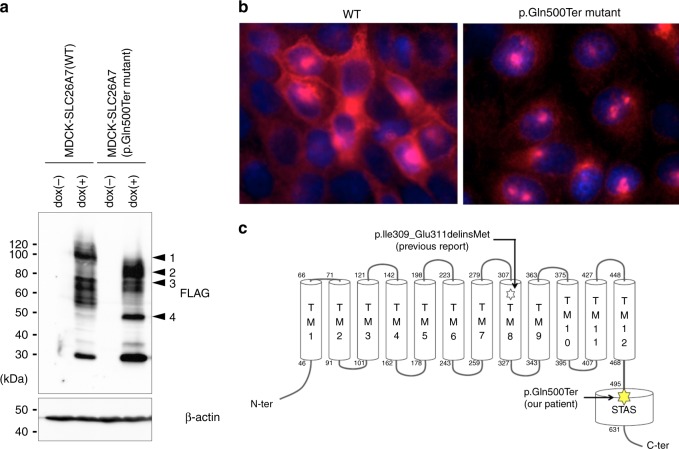
Localisation of the SLC26A7 p.Gln500Ter mutant. **a** Immunoblotting to confirm the FLAG-tagged SLC26A7 mutant in MDCK cells. β-actin served as an internal control; dox, doxycycline. Arrowheads indicate glycosylated wild type SLC26A7 (1), glycosylated SLC26A7 p.Gln500ter mutant (2), wild-type SLC26A7 (3), and SLC26A7 p.Gln500ter mutant (4). **b** Immunofluorescence analysis for FLAG (red) FLAG-tagged SLC26A7 mutant-transfected MDCK cells. Propidium iodide (blue) marks cell nuclei. **c** SLC26A7 protein structure and the positions of mutations in patients with congenital hypothyroidism. The SLC26A7 protein has 12 putative transmembrane and intracellular N- and C-terminal domains. The C-terminal cytoplasmic domain contains the STAS domain, which is shared among SLC26 family members. The mutations identified in our patients (filled star) and in the previously reported Arabian patients (open star) are illustrated. TM, transmembrane domain; STAS, sulphate transporters and anti-sigma factor antagonists

To analyse the consequences of the p.Gln500Ter mutation in *SLC26A7*, a FLAG-tagged expression vector was constructed and introduced into MDCK cells. As translation is interrupted by the p.Gln500Ter mutation, the mutant SLC26A7 displayed a smaller molecular weight band when compared to that in the wild type (WT), as assessed by western blot analysis (Fig. 5a). The uncropped blot images are shown in Supplementary Fig. 9. Although the localisation of the WT SLC26A7 was mainly on the cellular membrane, the mutant SLC26A7 tended to aggregate in the cytosol and not to be expressed on the cell membrane (Fig. 5b). Furthermore, we investigated the localisation of the p.Ile309_Glu311delinsMet mutant protein recently reported by Zou et al.^12^. Abnormal localisation as in our patients was demonstrated in the transiently transfected HEK293T cells (Supplementary Fig. 10), suggesting a reduction in function induced by both mutations. The p.Gln500Ter mutation in SLC26A7 is located at the N-terminus of the sulphate transporter in the anti-sigma factor antagonists (STAS) domain (Fig. 5c). Moreover, it has been reported that a defect in the STAS domain impairs membrane localisation for other members of the same gene family, *SLC26A3* and *SLC26A4*^13,14^. The impaired surface expression of SLC26A7 following a STAS domain mutation might represent a shared common characteristic among the proteins in this family.

### The SLC26A7 mutation abolishes the iodide transporter function

**Fig. 6:**
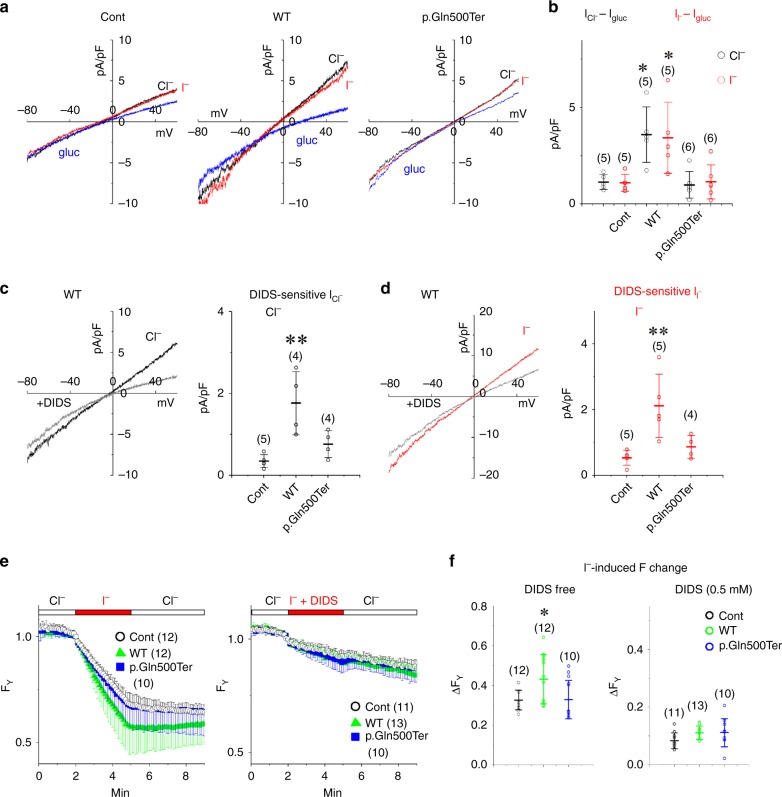
Electrophysiological and optophysiological analyses of the ion transport function of SLC26A7. WT, but not p.Gln500Ter SLC26A7 functions as an I^−^ transporter. **a** Current–voltage relationships. Shown are the current traces recorded during the ramp pulse protocol (−80 to 60 mV for 400 ms) from cells transfected with YFP alone (Cont) or with YFP and SLC26A7 constructs. The currents were recorded in different bath solutions (NaCl, black; NaI red; Na-glu, blue). **b** Summary of Cl^−^ and I^−^ current amplitudes (I_Cl_^−^–I_glu_ and I_I_^−^–I_glu_). Black and red circles represent the amplitudes of I_Cl_^−^–I_glu_ and I_I_^−^–I_glu_ at 29–31 mV in each cell, respectively. Numbers of cells are indicated in parentheses. *P < 0.05 (WT against control: *P* = 0.0130 (I_Cl_^−^–I_gl_) and 0.0207 (I_I_^−^–I_gl_), Tukey’s test). **c**, **d** Inhibition of the Cl^−^ and I^−^ current by DIDS. In the left panels, the Cl^−^ and I^−^ current traces (black and red lines) are shown in the presence or absence of 0.5 mM DIDS (grey lines). Black and red circles in the right panels show the amplitudes of the DIDS-sensitive I_Cl_^−^ and I_I_^−^ densities in each cell, respectively. Numbers of cells are indicated in parentheses. **P < 0.01 (WT against control: *P* = 0.0030 (NaCl) and 0.0055 (NaI), Tukey’s test). **e** The accumulation of I^−^. Shown are time lapse changes of the normalised fluorescence intensity of YFP-HQ/IL (F_Y_) in cells expressing the mutant YFP-HQ/IL alone (open circles, con) or in the presence of WT (green triangles) or mutant SLC26A7 (blue squares). The NaI bath solution was perfused for 3 min, as indicated by red bars on traces. DIDS (0.5 mM) was included in the NaI solution to inhibit the WT SLC26A7 channel (right panel). Numbers of cells are indicated in parentheses. **f** Summary of the F_Y_ change. Circles represent changes in F_Y_ at 3 min after the NaI perfusion. Numbers of cells are indicated in parentheses. *P < 0.05 (WT against control: *P* = 0.0288, Tukey’s test)

The mouse SLC26A7 was previously reported to serve as a Cl^−^ channel when expressed in *Xenopus* oocytes and HEK293 cells^9^. As we assumed that the human SLC26A7 functions as the I^−^ transporter, the macroscopic membrane current was recorded from cells expressing a successful YFP transfection maker. In HEK293T cells expressing YFP alone, a small outward current was recorded both in the NaCl and NaI bath solutions during the depolarising potential, which decreased in the Na-gluconate solution (Fig. 6a, b). Our results show that endogenous Cl^−^ channels/transporters are expressed in these cells at a low level. In cells co-transfected with WT SLC26A7, the amplitudes of the outward currents in the NaCl and NaI bath solutions were significantly increased (WT SLC26A7 against control: *P* = 0.0130 and 0.0207, respectively, Tukey’s test), while these increases were not observed in cells transfected with the p.Gln500Ter truncated mutant (Fig. 6a, b). In cells expressing WT SLC26A7, the application of a Cl^−^ channel blocker, 4,4ʹ-diisothiocyano-2,2ʹ-stilbenedisulphonic acid (DIDS) (0.5 mM for 20 s) decreased the amplitudes of the outward currents both in the NaCl and NaI bath solutions (Fig. 6c, d). The amplitude of the DIDS sensitive currents were larger in cells transfected with SLC26A7 than in those transfected with YFP alone (control) or with the p.Gln500Ter mutant and YFP (WT against control: *P* = 0.0030 (NaCl) and 0.0055 (NaI), Tukey’s test). These results suggest that WT SLC26A7 functions as a Cl^−^ and I^−^ transporter, and that the function is lost following the p.Gln500Ter mutation. The outward current in the NaI solution presumably reflects the I^−^ influx into the cell. We thus monitored the fluorescent intensity of the iodide sensitive YFP-HQ/IL (F_Y_). The perfusion of the NaI bath solution decreased the F_Y_ in cells expressing YFP-HQ/IL with or without SLC26A7 constructs, and these decreases were inhibited by DIDS (0.5 mM) when it was included in the NaI solution (Fig. 6e). Interestingly, WT SLC26A7 but not the p.Gln500Ter mutant or recently reported p.Ile309_Glu311delinsMet mutant^12^ accelerated the F_Y_ decrease (WT against control: *P* = 0.0288, Tukey’s test; WT against p.Ile309_Glu311delinsMet mutant: *P* = 0.0359, Tukey’s test; Fig. 6f and Supplementary Fig. 11), supporting the hypothesis that WT SLC26A7 functions as an I^−^ transporter whereas the p.Gln500Ter and p.Ile309_Glu311delinsMet mutants do not, although the activity of WT SLC26A7 may be lower than that of SLC26A4 (WT SLC26A7 against WT SLC26A4: *P* < 0.001, Tukey’s test; Supplementary Fig. 12).

## Discussion

To our knowledge, Pendred syndrome was first reported as a syndrome involving hearing loss and goitre by Dr. Vaughan Pendrin in 1986; the causative gene, *SLC26A4*, was discovered, and its iodide transport function was demonstrated in 1997–1999^15,16^. To date, SLC26A4 has been the only known exporter of iodide to the thyroid follicular lumen. However, humans may possess another iodide exporter, since the thyroid hormone can still be synthesised when the *SLC26A4* gene is non-functional^17^. Although the iodide transport functions of CLCN5 and CFTR have not been demonstrated experimentally, they are considered to be candidate thyroid iodide transporters, similar to SLC26A4^6,18^. Furthermore, the calcium-activated anion channel anoctamine 1 is expressed in the apical membrane of thyroid follicular cells and has iodide-transporting ability; thus, it is considered to contribute to iodide transport to the thyroid follicular lumen, along with SLC26A7^19^.

As first as we know, SLC26A7 was first discovered as a chloride/anion exchanger in the kidney and stomach, and a later study using *Xenopus* oocytes showed that SLC26A7 can also transport anions such as oxalate, sulphate, and iodide^9,20,21^. In this study, we examined the possibility that SLC26A7 may represent another iodide transporter aside from SLC26A4 in the thyroid gland. SLC26A7 is expressed predominantly on the luminal side of thyroid follicular cells and it possesses an iodide transport function. We also discovered a homozygous nonsense mutation in *SLC26A7* in two Japanese siblings with congenital hypothyroidism; the mutation abolished membrane localisation and the iodide transport function of SLC26A7. These results indicate that SLC26A7 is likely to be a new iodide transporter in the thyroid gland with the same function as SLC26A4.

Our results showed a slightly different result from that of Cangul et al.^8^. for the cellular localisation of SLC26A7. Cangul et al. produced a transgenic mouse line expressing FLAG-epitope-tagged SLC26A7 and reported that a positive signal for FLAG immunostaining was found in the intracellular region and areas close to the basal membrane. However, the positive signal was predominantly observed in the cells, and positive cells were restricted to a limited number of cells in a limited number follicles. Cangul et al. did not show double staining results with proteins expressed on the basal side of the cell; thus, further experiments are considered necessary to accurately ascertain the localisation of SLC26A7 expression. In our study, Fig. 3d also showed a different result from those of Gillam et al.^11^. For iodide transport experiments with cell culture inserts, the amount of iodide remaining in cells was reduced by the expression of SLC26A4 in the study of Gillam, but it did not change significantly in our study. The cause of this may be the difference in experimental systems such as the amount of radioactive NaI used, the method of gene transfer into the cells, and the difference in detection systems. In any case, careful re-examination in future studies is necessary.

*SLC26A7* and *SLC26A4* belong to the same SLC26 gene family^22^. The amino acid sequence homology between SLC26A7 and SC26A4 is the highest among SLC26A family genes, and both are highly expressed in the thyroid and kidney and function in chloride reabsorption, together with other SLC26A family members such as SLC26A3, A4, A6, A7, A9 and A11^23^. SLC26A7 is expressed in the kidney in the outer medullary collecting duct, whereas SLC26A4 is localised to the cortical collecting duct^21,24^. Although their expression patterns are completely different, these two regions are adjacent to each other in the collecting tube, and it is not unexpected for SLC26A7 and SLC26A4 to have a similar function. However, the transporter activity of SLC26A7 was significantly lower than that of SLC26A4. Due to the discrepancy between the lower iodide transport activity of SLC26A7 and the clinical severity of thyroid dysfunction in patients with a mutation in *SLC26A7* compared to those with Pendred syndrome, we speculate that SLC26A7 may be expressed more abundantly than SLC26A4 in the human thyroid gland or that SLC26A7 may have another function in addition to being an iodide transporter.

Pendred syndrome is an autosomal recessive disorder characterised by sensorineural hearing loss and hypothyroidism with goitre. Patients with Pendred syndrome develop goitre before puberty as a consequence of a partial organification defect. However, about 50% of patients with Pendred syndrome are euthyroid^19,25^, suggesting that SLC26A7 or another iodide transporter may compensate for the dysfunction of pendrin (SLC26A4). Using database analysis, Slc26a7 expression at the stria vascularis in *Slc26a4* (−/−) mice increased approximately sevenfold compared with that in *Slc26a4* (+/−) mice; thus, it is conceivable that increasing Slc26a7 expression in the stria vascularis in *Slc26a4* (−/−) mice may counteract *Slc26a4* loss^26^ (GSE10589 dataset). A similar compensation may occur in the thyroid. Thus, *Slc26a4* (−/−) mice displaying iodide deficiency are euthyroid, while *Slc26a7* (−/−) mice show hypothyroidism with histological thyroid abnormalities. In addition, the patients that presented with the *SLC26A7* mutation showed severe hypothyroidism, even though in Japan, dietary iodide is sufficient. Therefore, SLC26A7 may be an indispensable iodide transporter in the thyroid. Moreover, when SLC26A4 does not function properly, SLC26A7 may function as an iodide transporter to the follicular lumen thus replacing SLC26A4.

Recently, Arabian, Pakistani, and Turkish sibling cases with congenital hypothyroidism were reported to present with a homozygous mutation in *SLC26A7*^8,12^. In the Arabian case, identified mutations were described as c.927_930del; p.Ile309Metfs*9 and c.932_933del; p.Glu311Glyfs*28 and were claimed to represent a loss of function. However, according to the electropherograms, the mutation should be designated as c.927_933delinsG; p.Ile309_Glu311delinsMet and causes a 6-bp in-frame deletion. Therefore, its pathogenicity needs to be assessed using functional studies. As the mutation causes the deletion of two amino acid residues located in the putative transmembrane domain, it may be pathogenic, as suggested by several prediction methods (Mutation Taster, disease causing; PROVEAN, deleterious). To test the functional consequence of the mutation, we inserted c.927_933delinsG into a FLAG-tagged SLC26A7 expression vector. The mutated SLC26A7 (p.Ile309_Glu311delinsMet) clearly showed an abnormal protein distribution (Supplementary Fig. 10). More importantly, the mutated SLC26A7 (p.Ile309_Glu311delinsMet) showed an impaired iodide transport function (Supplementary Fig. 11). Notably, the reported Arabian siblings showed similar clinical characteristics to our patients. Namely, they exhibited congenital hypothyroidism without other complications, and one of the patients had goitre (Table 3). Therefore, we propose that biallelic loss-of-function mutations in *SLC26A7* cause congenital goitrous hypothyroidism. Furthermore, no patients showed hearing loss or kidney dysfunction. Since SLC26A7 is not expressed in the cochlea, the absence of hearing complications is understandable. However, as SLC26A7 is expressed in the kidney, a careful observation of the renal function in patients with *SLC26A7* mutations is needed. Furthermore, because the clinical features are masked by the administration of thyroid hormones, patients with a mutation in *SLC26A7* are likely undiagnosed patients with congenital hypothyroidism.

**Table 3:** Clinical features of patients with the SLC26A7 mutations and with the SLC26A4 mutations (Pendred syndrome)

	*SLC26A7*				Pendred syndrome (*SLC26A4*)
	Our study		Previous report		
	Patient 1	Patient 2	Elder sister (43.1)	Younger sister (43.2)	
Mutation	c.1498C > T		c.927_933delinsG		
Zygosity	Homozygous		Homozygous		
Amino acid change	p.Gln500Ter		p.Ile309_Glu311delinsMet		
Thyroid					
Hypothyroidism*	Yes	Yes	Yes	Yes	Euthyroid in most patients
Goitre	Yes	Yes	Yes	No	50–83% of the patients
Severity of hypothyroidism	Moderate	Severe	N.A.	N.A.	N.A.
Age at diagnosis of hypothyroidism	15 days	5 years	3 years	N.A.	
High level of serum TG	Yes	Yes	N.A.	N.A.	Yes
Other Feature					
Renal tubular acidosis*	No	No	No	No	N.A.
Deafness	No	No	N.A.	N.A.	Yes
EVA	N.A.	N.A.	N.A.	N.A.	80–100% of the patients
Impairment of gastric acid secretion*	N.A.	N.A.	N.A.	N.A.	N.A.

*TG,* thyroglobulin; *EVA,* enlarged vertibular aqueduct; *N.A.,* not available

^*^Phenotype of Slc26a7 knock out mice

The homozygous mutations in *SLC26A7* identified so far disrupt the STAS domain^27–29^. This domain is present from eubacteria onwards as a phylogenetically conserved fold encoded by highly divergent amino acid sequences. Consistent with previous studies suggesting that mutations in the STAS domain impair the cell surface expression and function of the SLC26 transporter^13,14,30^, the *SLC26A7* mutation identified in this study led to the abnormal localisation (Fig. 5b and Supplementary Fig. 10) and dysfunction of this protein (Fig. 6 and Supplementary Fig. 11). However, the possibility of nonsense-mediated decay being involved in the associated dysfunction cannot be ruled out, and thus, further study is considered necessary.

In conclusion, we demonstrate that SLC26A7 may be a novel iodide transporter in the thyroid and that its dysfunction causes congenital goitrous hypothyroidism.

## Methods

### Immunofluorescence staining and immunohistochemistry

Immunofluorescence (IF) histochemistry was conducted using formalin-fixed, paraffin-embedded human thyroid tissues which were collected at the Kyorin University Hospital. This study was approved by the Institutional Ethics Board of Kyorin University School of Medicine (approval number 257). Consecutive tissue sections, each 4 μm in thickness, were immunostained using rabbit anti-SLC26A7 (1:1000; SCRUM; Tokyo, Japan), mouse anti-SLC5A5 (1:1000; ab17795; Abcam, Cambridge, UK) or rabbit anti-SLC26A4 antibodies (1:500; ab98091; Abcam). After antigen retrieval treatment (0.1 mol/L sodium citrate buffer, pH 6.0, at 121 °C for 5 min), a mixture of a mouse monoclonal anti-SLC5A5 antibody and rabbit polyclonal anti-SLC26A7 or SLC26A4 antibody was applied to the tissue section-mounted slides, which were then incubated overnight at 4 °C. After being thoroughly washed with 15 mmol/L phosphate-buffered saline (PBS) (pH 7.4), the slides were incubated with a mixture of an Alexa Fluor 488-labelled anti-rabbit secondary antibody (1:500; 4412; Cell Signalling Technology, Beverly, MA, USA) and an Alexa Fluor 594-labelled anti-mouse secondary antibody (1:500; 8890; Cell Signalling Technology) for 1 h at room temperature. After thorough washes with PBS, the nuclei were counterstained with DAPI. Analyses were performed using a fluorescence microscope (Olympus, Tokyo, Japan).

For immunohistochemistry (IHC), formalin-fixed, paraffin-embedded tissue sections from ten thyroid tissues were stained with haematoxylin and eosin or were immunostained using rabbit anti-SLC26A7, mouse anti-SLC5A5 or rabbit anti-SLC26A4 antibodies. The endogenous peroxidase activity was blocked with a 0.3% H_2_O_2_-methanol solution for 15 min at room temperature. After antigen retrieval treatment described above, IHC was performed using an Envision FLEX detection system (DakoCytomation, Glostrup, Denmark) according to the manufacturer’s instructions. Peroxidase activity was visualised with 3,3ʹ-diaminobenzidine (DakoCytomation).

For IF cytochemistry, cultured cells on glass slides or cell culture inserts were washed twice with 15 mmol/L PBS (pH 7.4) and were then fixed with formalin at room temperature for 15 min. After cellular permeabilisation following incubation for 10 min at 4 °C with PBS containing 0.1% Triton X-100, mouse anti-FLAG (1:100; F1804, Sigma-Aldrich, St. Louis, MO) or rabbit anti-sodium potassium ATPase (1:10000; ab76020, Abcam) antibodies were applied to the fixed cells and incubated overnight at 4 °C. After being thoroughly washed with PBS, the fixed cells were incubated with a mixture of an Alexa Fluor 647-labelled anti-mouse secondary antibody (1:500; A-21235; Invitrogen; Carlsbad, CA) and an Alexa Fluor 488-labelled anti-rabbit secondary antibody (1:500; A-11008; Invitrogen). After thorough washes with PBS, the nuclei were counterstained with 4ʹ,6-diamidino-2-phenylindole (DAPI). The analyses were performed using a fluorescence microscope (Olympus, Tokyo, Japan) and confocal microscopy (Fluoview FV1000, Olympus).

For analysing the cellular localisation of both HeLa cells stably expressing WT SLC26A7 and HEK293T cells transiently transfected with WT and mutant SLC26A7, cells were washed twice with PBS and then fixed with 4% paraformaldehyde at room temperature for 10 min. After cellular permeabilisation following incubation for 10 min at room temperature with PBS containing 0.2% Tween 20, the cells were blocked with Blocking one (03953-66; Nacalai Tesque) at room temperature for 1 h and incubated with mixture of a mouse anti-FLAG (0.25 µg/mL; A00187-100; GenScript) and a rabbit anti-integrin alpha 5 (ITGA5) (1:250; ab150361; Abcam) antibodies at room temperature for 1 h. After being thoroughly washed with PBS, the cells were incubated with a mixture of an Alexa Fluor 488-labelled anti-mouse secondary antibody (1:200; ab150109; Abcam) and an Alexa Fluor 594-labelled anti-rabbit secondary antibody (1:500; ab150068; Abcam) at room temperature for 30 min. After thorough washes with PBS, the nuclei were counterstained with DAPI. The analyses were performed using a confocal microscopy (Nikon-A1R; Nikon).

### Cell culture and gene transfection

A canine kidney cell line (Madin-Darby Canine Kidney II; MDCK), an immortalised normal thyroid follicular cell line (Nthy ori-3; Nthy), a monkey kidney cell line (COS-7), a human embryonic kidney cell line (HEK293T and HEK-GP2-293), a human thyroid cancer cell line (WRO), and a human cervix adenocarcinoma cell line (HeLa) were used in this study. MDCK and Nthy were purchased from the European Collection of Authenticated Cell Cultures (catalogue numbers 00062107 and 90011609 respectively; Salisbury, UK). COS-7 and HeLa cells were purchased from the American Type Culture Collection (catalogue numbers CRL-1651 and CCL-2, respectively; Manassas, VA). HEK293T cells were purchased from RIKEN Cell Bank (catalogue number RCB2202; Tsukuba, Japan). HEK-GP2–293 cell line was purchased from Clontech (Mountain View, CA). All cells were cultured in DMEM supplemented with 10% heat-inactivated foetal calf serum, 100 U/mL penicillin and 100 mg/mL streptomycin. The cells were maintained at 37 °C in 5% CO_2_. To analyse SLC26A7 function and compare it with that of SLC26A4, SLC26A7, SLC26A4 or SLC5A5-transfected cells were established. SLC26A7, SLC26A4 and SLC5A5 cDNA was synthesised from total RNA from normal thyroid tissue using the SuperScript first-strand synthesis system according to the manufacturer’s instructions (Life Technologies, Carlsbad, CA), amplified by PCR using a specific primer set and the primeSTAR GXL DNA polymerase (Takara, Shiga, Japan), and sequenced. Primer sequences used for amplifying coding sequence (CDS) of SLC26A7, SLC26A4 and SLC5A5 were listed in Table 4. The SLC26A7 CDS containing a FLAG-tag sequence or the SLC26A4 CDS were inserted into the pRetroX-tight-Pur vector (Clontech). The SLC5A5 CDS was inserted into the pQCXIH vector (Clontech). The mutant construct in our patient (c.1498 C > T) was created by site directed mutagenesis using the KOD-Plus-Mutagenesis Kit (Toyobo, Osaka, Japan). Primer sequences used for amplifying the SLC26A7 c.1498 C > T mutant and c.927_933delinsG mutant were listed in Table 4. The constructed vector was transfected into MDCK, Nthy and COS-7 cells using a retroviral expression system (Clontech). After 3 weeks of selection with puromycin (Clontech; 1 μg/mL, for SLC26A7 or SLC26A4) or hygromycin b (FUJIFILM Wako Pure Chemical Industries, Ltd., Osaka, Japan; 500 μg/mL, for SLC5A5), SLC26A7 or SLC26A4 was induced in the transfected cells by supplementing the culture medium with doxycycline (Dox, Clontech; 1 μg/mL) and incubating the cells for 24 h, unless otherwise specified.

**Table 4:** Primer sets used in this study

Primer Name	Primer sequences	Product size (bp)
SLC26A7 (for construction of expression vector)	F-5ʹ-TGAAAGGAGGTGTTCTGCAA-3ʹ	2099
	R-5ʹ-TATGGCCTCTTCAGGCAGTT-3ʹ	
SLC26A4 (for construction of expression vector)	F-5ʹ-GTCCCACTGCCTTCTGAGAG-3ʹ	2785
	R-5ʹ-TCATTCGTGACTCGCTTGAC-3ʹ	
SLC5A5 (for construction of expression vector)	F-5ʹ-CCTCATGGAGGCCGTGGAGACC-3ʹ	1943
	R-5ʹ-CCTGTCCTCAGAGGTTTGTCTCCTGC-3ʹ	
SLC26A7 (for c.1498C > T mutant)	F-5ʹ-TAGCAGGTGAAAATTATCTCAATAA-3ʹ	
	R-5ʹ-CAGGGTTTCACTGTCCATTTCTGTC-3ʹ	
SLC26A7 (for c.927_933delinsG mutant)	F-5′-GGCTTTCGGAGTGGCACTTGTAGGCT-3′	
	R-5′-ATCACCGCAGAGAGGATGTTCATCG-3′	
SLC26A7 (for qRT-PCR)	F-5ʹ-GGCACTTGTAGGCTATGTGGC-3ʹ	150
	R-5ʹ-GCAGCAGCACTTGGTATGCA-3ʹ	
SLC26A7 (for RT-PCR)	F-5ʹ-CACTGTGTCTGGGATAATGTTGG-3ʹ	353
	R-5ʹ-CCAGTTGCAGCACAAACATG-3ʹ	
SLC26A4 (for qRT-PCR)	F-5ʹ-CGGATATGGTCTCTACTCTGC-3ʹ	161
	R-5ʹ-TGCTGCTGGATACGAGAAAGTG-3ʹ	
SLC5A5 (for qRT-PCR)	F-5ʹ-TGCCCTCTCTGAGCCTCAAT-3ʹ	62
	R-5ʹ-CCTGATCACAGCTGTCACTGTCT-3ʹ	
RPS18 (for qRT-PCR)	F-5ʹ-TTTGCGAGTACTCAACACCAACATC-3ʹ	89
	R-5ʹ-GAGCATATCTTCGGCCCACAC-3ʹ	
GAPDH (for RT-PCR)	F-5ʹ-GCACCGTCAAGGCTGAGAAC-3ʹ	138
	R-5ʹ-TGGTGAAGACGCCAGTGGA-3ʹ	

*qRT-PCR* quantitative RT-PCR, *F* forward primer sequence, *R* reverse primer sequence

For the generation of stable HeLa expression cells, pCAGGS-Neo-SL26A7 WT was transfected into Hela cells (50–80% confluency) grown in Dulbecco’s modified Eagle’s medium (Gibco, Gaithersburg, MD) supplemented with 10% foetal bovine serum (Gibco) in a 6-well plate using Lipofectamine 3000 (Invitrogen) according to the manufacturer’s protocol. At 48 h post-transfection, G418 (Gibco) was added to the medium at a final concentration of 800 µg/mL to select clones carrying stably integrated plasmid DNA. The medium was changed every 3 days. Three weeks later, G418-resistant colonies was selected and expanded into cell lines for further analysis.

### Semiquantitative RT-PCR (qRT-PCR) and RT-PCR

Total RNA was extracted from formalin-fixed, paraffin-embedded tissue sections using the RNase-free deparaffinisation solution (Qiagen, Hilden, Germany) and the RNeasy FFPE kit (Qiagen), and from the cell lines using TRIzol (Life Technologies) according to the manufacturer’s instructions. First-strand complementary cDNA was synthesised from the total RNA using the SuperScript First-Strand Synthesis System (Invitrogen). The resulting cDNAs were used as templates in real-time PCR with the Fast SYBR Green master mix (number 4385612; Applied Biosystems, Waltham, MA) or in RT-PCR with GoTaq Green Master Mix (Promega, Madison, WI). The reactions were run on a StepOnePlus real-time PCR system (Applied Biosystems) or a thermal cycler. Primer sequences used for amplifying SLC26A7 (qRT-PCR), SLC26A7 (RT-PCR)^31^, SLC26A4^32^ SLC5A5, RPS18, and GAPDH were listed in Table 4. The RPS18 and GAPDH were used for each sample as internal controls.

### SDS-PAGE and western blotting

Membrane protein-enriched samples from cultured cells were used for loading, as the iodide transporter normally functions within the cell membrane. After a PBS wash, the cells were harvested using an elution buffer (20 mmol/L HEPES, pH 7.6, 20 mmol/L NaCl, 0.5 mmol/L EDTA, 10% glycerol) and scraped into a chilled microtube. The cells were sonicated and centrifuged for 30 min at 10,000*g* at 4 °C, and the pellet was resuspended using a membrane-solubilizing buffer (elution buffer with 1% triton X-100) and incubated on ice for 30 min. After centrifugation, the supernatant was used as the membrane protein-enriched sample. The sample were separated by 8% sodium dodecyl sulfate polyacrylamide gel electrophoresis and transferred to poly vinylidene fluoride membrane. These membranes were blocked for 30 min at room temperature with 0.5% skim milk in PBS containing 0.1% (v/v) Tween 20 (PBS-T) and then incubated with appropriately diluted rabbit anti-SLC26A7 (1:1000; BMP084, MBL), rabbit anti-SLC26A4 (1:500; ab98091, Abcam), mouse anti- SLC5A5 (1:1000; ab17795, Abcam), or mouse anti-FLAG antibodies (1:1000; F1840, Sigma-Aldrich) as primary antibodies. A rabbit anti-sodium potassium ATPase antibody (1:10000; ab76020, Abcam) and a mouse anti-β-actin (1:10000; A5441; Sigma-Aldrich) antibody were used as internal controls.

### Iodide transport assay

Cells were split into 24-well plates and grown to 80% confluency. Iodide transport assays were performed 24 h after the induction of the transgene using doxycycline. Cells were washed three times with pre-warmed Hank's balanced salt solution (HBSS, Nacalai Tesque, Kyoto, Japan). Next, the cells were incubated with uptake solution containing 5 nM of radioactive NaI and 10 µM cold NaI. The cells were incubated for 5 min at 37 °C and substrate uptake was terminated by washing the cells with ice-cold uptake solution three times. Cells were solubilised in 0.1 N sodium hydroxide and the radioactivity in the cell lysates was measured using a Typhoon FLA9500 system (GE Healthcare, Chicago, IL).

To measure iodide transport from the bottom to the upper chamber, we used a modified Gillam MP method as a reference^11^, the method is as follows. Cells were grown on culture inserts (Corning, NY) until the formation of a monolayer. Next, the electric resistance was measured with an EVOM2 Epithelial Voltohmmeter (LMS, Tokyo, Japan) to ensure the formation of an intact polarised monolayer. Cell culture inserts with a transepithelial electrical resistance (Ω) <300 were determined to have tight junction formation and were used for the experiment. Cells were washed with HBSS solution and incubated at 37 °C with HBSS containing 2.5 nM radioactive NaI and 10 µM cold NaI in the bottom wells. After a 45-min incubation, the radioactivity on the opposite side and the intracellular radioactivity was considered to represent the transcellular movement of iodide.

### Whole-exome sequencing analysis

For genetic analyses, we obtained peripheral blood samples from the affected siblings, an unaffected sibling, and their parents by venepuncture. All patients and their parents provided written informed consent. The Institutional Review Board of the Nagoya City University Graduate School of Medical Sciences approved the study (approval no. 187). Genomic DNA was extracted from peripheral-blood samples using the QIAamp Blood Midi Kit (Qiagen).

A total amount of 1.0 μg of genomic DNA per sample was used for the DNA sample preparation. Sequencing libraries were generated using the Agilent SureSelect Human All ExonV6 kit (Agilent Technologies, Santa Clara, CA) following the manufacturer’s recommendations. Briefly, fragmentation was carried out using a hydrodynamic shearing system (Covaris, Woburn, MS) to generate 180–280 bp fragments, and adaptor oligonucleotides were ligated. The libraries were sequenced on HiSeq4000 sequencers.

### Sequence data analysis and validation

Sequence data were analysed using the CLC Genomics Workbench version 8.0 (CLC bio, Aarhus, Denmark). Variants detected by whole-exome sequencing were validated by conventional Sanger sequencing. We referred to the ExAC (http://exac.broadinstitute.org) and HGVD datasets (http://www.hgvd.genome.med.kyoto-u.ac.jp.med.kyoto-u.ac.jp) and excluded common polymorphisms whose allele frequencies in the general population were above 0.01. We followed the American College of Medical Genetics and Genomics standards and guidelines to determine variant classification (pathogenic, likely pathogenic, uncertain significance, likely benign or benign).

### Electrophysiology

HEK293T cells were seeded on poly-l-lysine coated glass and transfected with plasmid cDNAs encoding YFP with or without SLC26A7 constructs by using Lipofectamine 2000 (Invitrogen). Electrophysiological and optical experiments were performed 36–48 h after the transfection.

The macroscopic current was recorded from isolated single cells expressing a YFP marker indicating a successful transfection at room temperature, using the whole cell patch clamp technique, Axopatch 200B amplifiers, Digidata 1322A, and the pClamp 9 software (Molecular Devices, San Jose, CA), as previously described^33^. The NaCl bath solution contained 140 mM NaCl, 4 mM KCl, 10 mM HEPES, 1 mM CaCl_2_, and 0.3 mM MgCl_2_ (pH = 7.4). In the current recording, KCl was excluded from bath solution and the 140 mM NaCl was substituted with 140 mM NaI or 140 mM Na-gluconate (NaI or Na-glu bath solution, respectively). The pipette solution contained 130 mM N-methyl-d-glucamine (NMDG)-Cl, 10 mM HEPES, 4 mM MgCl_2_, and 10 mM EGTA, (pH = 7.3). An Ag–AgCl wire was grounded through an agar bridge (3 M KCl), as the reference electrode. After establishing the whole cell configuration, cells were held at 0 mV and ramp pulses (from −80 to 60 mV for 400 ms) were applied every 5 seconds. Cells were continuously perfused with the NaCl bath solution and the replacement of the major anion was performed by exchanging the NaCl solution for the NaI or Na-glu bath solution. The average amplitude of the 29–31 mV (I) currents recorded in each bath solution was measured. The I in the Na-glu or bath solutions including 0.5 mM DIDS was subtracted from I in the NaCl (I_Cl_^−^) or NaI bath solution (I_I_^−^). To minimise differences in cell size, I was normalised by the membrane capacitance.

### Generation of iodide-sensitive YFP-HQ/IL

Using PCR, two mutations (His148Gln/Ile152Leu) were introduced into YFP to generate the iodide-sensitive YFP (YFP-HQ/IL)^33,34^; primers were designed to introduce site-directed mutations.

### Imaging

Optical experiments were performed with an inverted microscope (Olympus) and a cooled CCD camera (RETIGA 6000, QImaging), as previously described^35^. The excitation of the YFP-HQ/IL by a 515 nm laser line and the image acquisition were controlled by using the MetaFluor imaging software (Molecular Devices). Fluorescence images of YFP-HQ/IL were acquired every 6 s and the fluorescence intensities (F_Y_) were measured from each cell expressing YFP-HQ/IL by subtracting the cell free background intensity. The accumulation of iodide in the cytosol was initiated by exchanging the perfusion solution from NaCl to NaI. The decrease in F_Y_ at 3 min after the NaI perfusion was normalised to the basal F_Y_ at 0 min following the NaI perfusion.

### Statistical analysis

All averaged data are presented as the mean ± SD. The *n* value indicates the number of current recordings or the number of cells for imaging. The statistical significance between two groups was estimated using a Student’s *t* test, and between three groups was estimated using a one-way analysis of variance followed by Tukey’s test; *P* < 0.05 was considered statistically significant. All experiments were confirmed the reproducibility.

### Reporting summary

Further information on research design is available in the Nature Research Reporting Summary linked to this article.

## Supplementary information

Description of Supplementary Data

Reporting Summary

Supplementary Data 1

Supplementary Information

## Data Availability

The whole-exome sequencing data reported in this study are available in the Human Genetic Variation Database (http://www.hgvd.genome.med.kyoto-u.ac.jp/repository/HGV0000010.html) with Accession ID HGV0000010. Due to privacy issues, approval for data access must be obtained from one of the corresponding authors. Other datasets generated during and/or analysed during the current study are available from the corresponding author on reasonable request.
