# Supplementary material for: Congenital goitrous hypothyroidism is caused by dysfunction of the iodide transporter SLC26A7
Source: Commun Biol. 2019 Jul 24;2:270. doi: 10.1038/s42003-019-0503-6 (PMC6656751; doi:10.1038/s42003-019-0503-6)
Supplement: Supplementary file 1 — Description of Supplementary Data [file 42003_2019_503_MOESM1_ESM.docx]

**Description of Additional Supplementary Files**

**File Name**: Supplementary Data 1

**Description**: Source data
