# Supplementary material for: Congenital goitrous hypothyroidism is caused by dysfunction of the iodide transporter SLC26A7
Source: Commun Biol. 2019 Jul 24;2:270. doi: 10.1038/s42003-019-0503-6 (PMC6656751; doi:10.1038/s42003-019-0503-6)
Supplement: Supplementary file 2 — Reporting Summary [file 42003_2019_503_MOESM2_ESM.pdf]

nature research

Corresponding author(s): Hiroshi Kamma
Department of Pathology, Kyorin University School of Medicine, Tokyo, Japan
Postal code: 181-8611
Phone: +81-422-47-5511
Fax: +81-422-40-7093
Email address: hkamma@ks.kyorin-u.ac.jp

Haruo Mizuno
Department of Pediatrics, International University of Health and Welfare, School of Medicine, Narita, Japan
Postal code: 286-8686
Phone: +81-287-39-3060
Fax: +81-287-39-3001
Email address: hamizuno@iuhw.ac.jp

Last updated by author(s): May 24, 2019

Reporting Summary

Nature Research wishes to improve the reproducibility of the work that we publish. This form provides structure for consistency and transparency in reporting. For further information on Nature Research policies, see Authors & Referees and the Editorial Policy Checklist.

Statistics

For all statistical analyses, confirm that the following items are present in the figure legend, table legend, main text, or Methods section.

n/a	Confirmed	
☐	☒	The exact sample size (*n*) for each experimental group/condition, given as a discrete number and unit of measurement
☐	☒	A statement on whether measurements were taken from distinct samples or whether the same sample was measured repeatedly
☐	☒	The statistical test(s) used AND whether they are one- or two-sided<br>*Only common tests should be described solely by name; describe more complex techniques in the Methods section.*
☒	☐	A description of all covariates tested
☒	☐	A description of any assumptions or corrections, such as tests of normality and adjustment for multiple comparisons
☒	☐	A full description of the statistical parameters including central tendency (e.g. means) or other basic estimates (e.g. regression coefficient) AND variation (e.g. standard deviation) or associated estimates of uncertainty (e.g. confidence intervals)
☐	☒	For null hypothesis testing, the test statistic (e.g. *F*, *t*, *r*) with confidence intervals, effect sizes, degrees of freedom and *P* value noted<br>*Give P values as exact values whenever suitable.*
☒	☐	For Bayesian analysis, information on the choice of priors and Markov chain Monte Carlo settings
☒	☐	For hierarchical and complex designs, identification of the appropriate level for tests and full reporting of outcomes
☒	☐	Estimates of effect sizes (e.g. Cohen's *d*, Pearson's *r*), indicating how they were calculated

Our web collection on statistics for biologists contains articles on many of the points above.

Software and code

Policy information about availability of computer code

Data collection	No software was used.
Data analysis	No software was used.

For manuscripts utilizing custom algorithms or software that are central to the research but not yet described in published literature, software must be made available to editors/reviewers. We strongly encourage code deposition in a community repository (e.g. GitHub). See the Nature Research guidelines for submitting code & software for further information.

Data

Policy information about availability of data

All manuscripts must include a data availability statement. This statement should provide the following information, where applicable:

- Accession codes, unique identifiers, or web links for publicly available datasets
- A list of figures that have associated raw data
- A description of any restrictions on data availability

Data availability statement was included the manuscript.

Field-specific reporting

Please select the one below that is the best fit for your research. If you are not sure, read the appropriate sections before making your selection.

☒ Life sciences ☐ Behavioural & social sciences ☐ Ecological, evolutionary & environmental sciences

For a reference copy of the document with all sections, see nature.com/documents/nr-reporting-summary-flat.pdf

Life sciences study design

All studies must disclose on these points even when the disclosure is negative.

Sample size	Sample sizes were chosen by consideration that it is sufficient to find a trend of data.
Data exclusions	No data were excluded.
Replication	All attempts at replication were successful and we confirmed it.
Randomization	Randomization was considered not essential for this study.
Blinding	Blinding was considered not essential for this study.

Reporting for specific materials, systems and methods

We require information from authors about some types of materials, experimental systems and methods used in many studies. Here, indicate whether each material, system or method listed is relevant to your study. If you are not sure if a list item applies to your research, read the appropriate section before selecting a response.

Materials & experimental systems

n/a	Involved in the study	
☐	☒	Antibodies
☐	☒	Eukaryotic cell lines
☒	☐	Palaeontology
☒	☐	Animals and other organisms
☒	☐	Human research participants
☒	☐	Clinical data

Methods

n/a	Involved in the study	
☒	☐	ChIP-seq
☒	☐	Flow cytometry
☒	☐	MRI-based neuroimaging

Antibodies

Antibodies used	Rabbit anti-SLC26A7 antibody (MBLMEDICAL and BIOLOGICAL LABORATORIES, BMP084), rabbit anti-SLC26A7 (SCRUM, custom antibody), rabbit anti-SLC26A4 antibody (Abcam, ab98091), mouse anti-SLC5A5 antibody (Abcam, ab17795), mouse anti-FLAG antibody (Sigma-Aldrich, F1840), mouse anti-FLAG (GenScript, A00187-100), rabbit anti-sodium potassium ATPase antibody (Abcam, ab76020), mouse anti-β-actin antibody (Sigma-Aldrich, A5441), rabbit anti-integrin alpha 5 antibody (Abcam, ab150361) were used in this study.
Validation	All antibodies in this study were validated by the preliminary experiment.

Eukaryotic cell lines

Policy information about cell lines

Cell line source(s)	Madin-Darby Canine Kidney II (MDCK) and normal thyroid follicular cell line (Nthy ori-3) were purchased from the European

Cell line source(s)	Collection of Authenticated Cell Cultures (catalogue number 00062107 and 90011609 respectively). Monkey kidney cell line (COS-7) and human cervix adenocarcinoma cell line (HeLa) were purchased from the American Type Culture Collection (catalogue number CRL-1651 and CCL-2, respectively). Human embryonic kidney cell line (HEK-GP2-293) was purchased from Clontech (catalogue number 631530). Human embryonic kidney cell line (HEK293T) was purchased from RIKEN Cell Bank (catalogue number RCB2202).
Authentication	None of the cell lines used were authenticated.
Mycoplasma contamination	The cell lines were not tested for mycoplasma contamination.
Commonly misidentified lines (See ICLAC register)	There were no commonly misidentified cell lines used in the study.
