# Supplementary material for: Congenital goitrous hypothyroidism is caused by dysfunction of the iodide transporter SLC26A7
Source: Commun Biol. 2019 Jul 24;2:270. doi: 10.1038/s42003-019-0503-6 (PMC6656751; doi:10.1038/s42003-019-0503-6)
Supplement: Supplementary file 4 — Supplementary Information [file 42003_2019_503_MOESM4_ESM.pdf]

Supplementary Figure 1

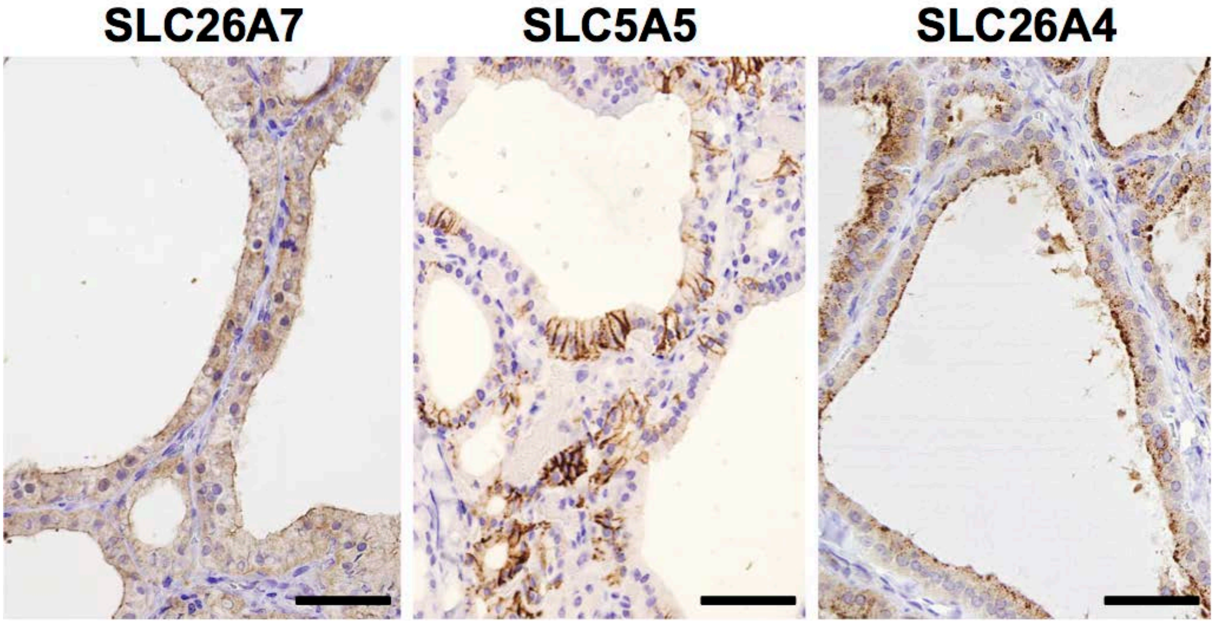

Supplementary Figure 1. Immunohistochemical staining of SLC26A7 (left), SLC5A5 (centre), and SLC26A4 (right) in thyroid tissues. Scale bar, 50 μm.

Supplementary Figure 2

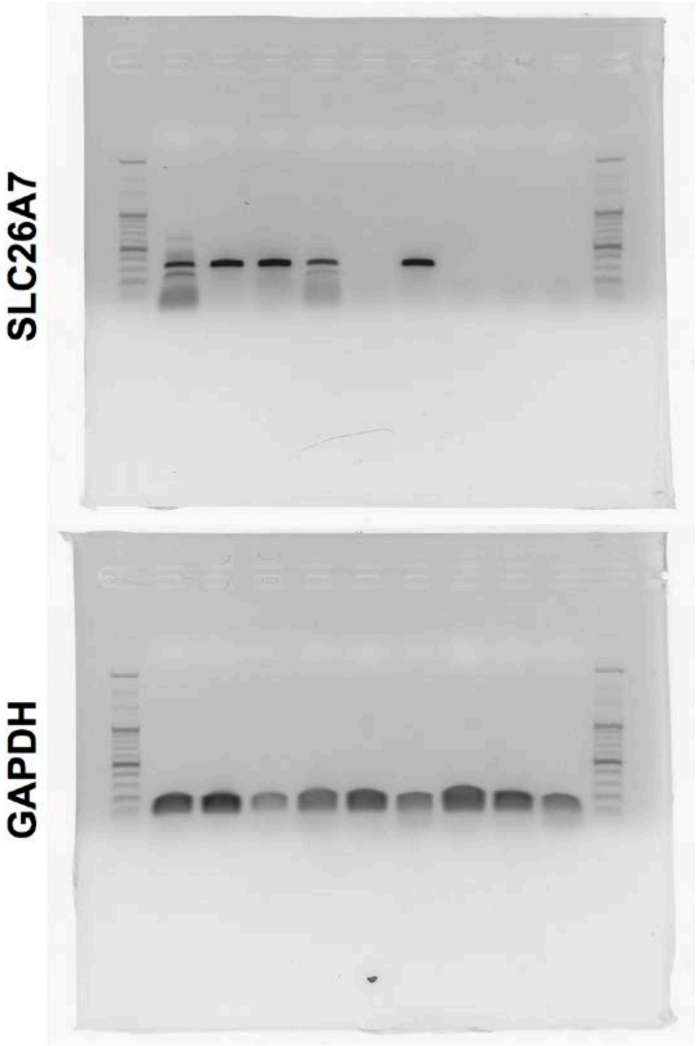

Supplementary Figure 2. Uncropped gel image in Figure 1b.

Supplementary Figure 3

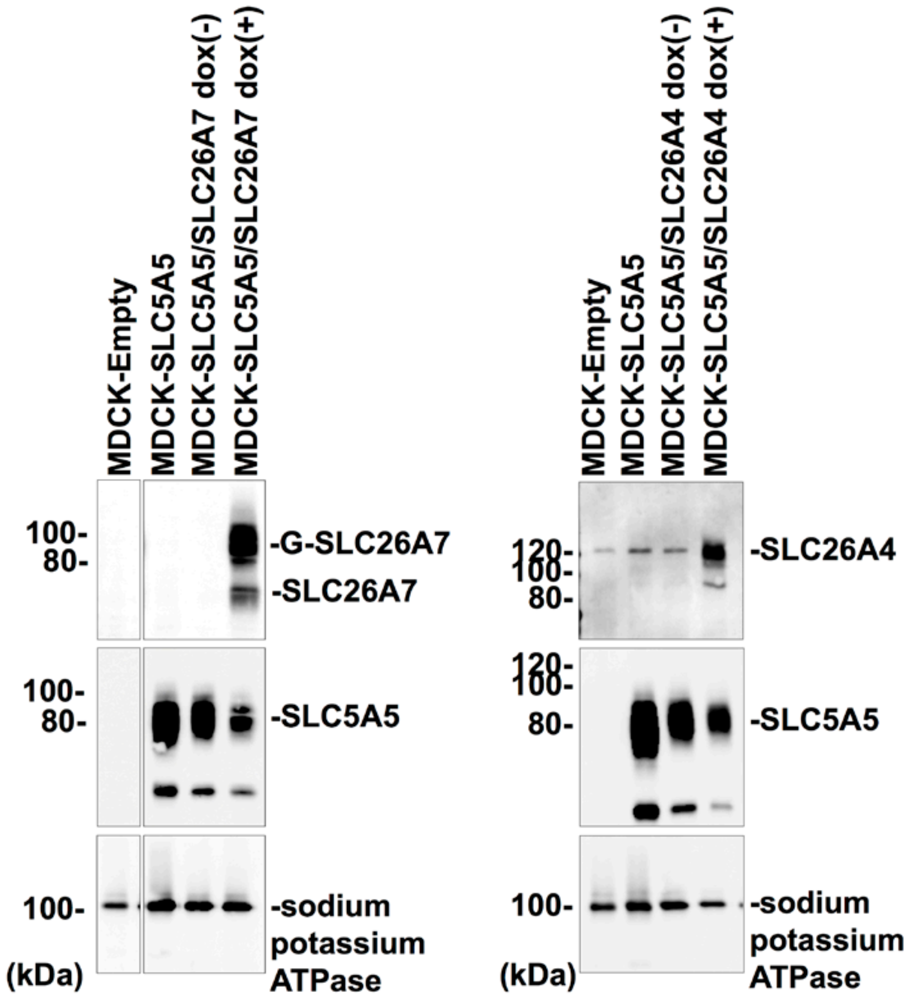

Supplementary Figure 3. Immunoblot analysis to confirm the gene transduction of *SLC26A7* and *SLC26A4* and *SLC5A5* in MDCK cells. Sodium potassium ATPase served as an internal control. dox, doxycycline; G-SLC26A7, glycosylated SLC26A7.

Supplementary Figure 4

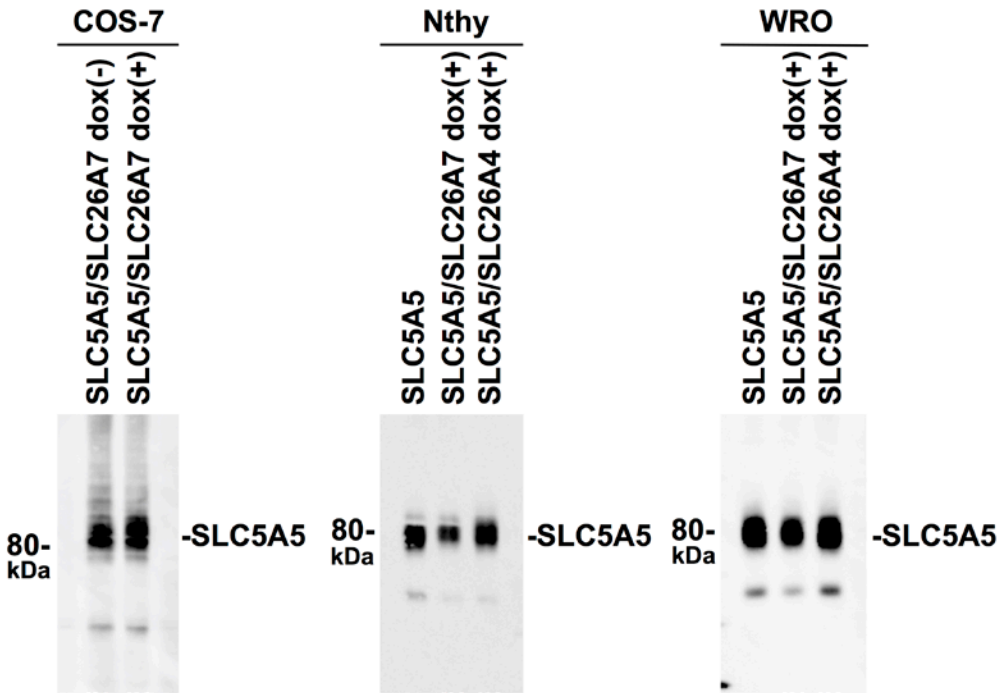

Supplementary Figure 4. Immunoblot analysis of SLC5A5 in SLC5A5 or SLC5A5 and SLC26A7 (SLC5A5/SLC26A7), or SLC5A5 and SLC26A4 (SLC5A5/ SLC26A4) in inducible COS-7, Nthy and WRO cells in the absence or presence of the inducer doxycycline (dox(-), dox(+) respectively).

Supplementary Figure 5

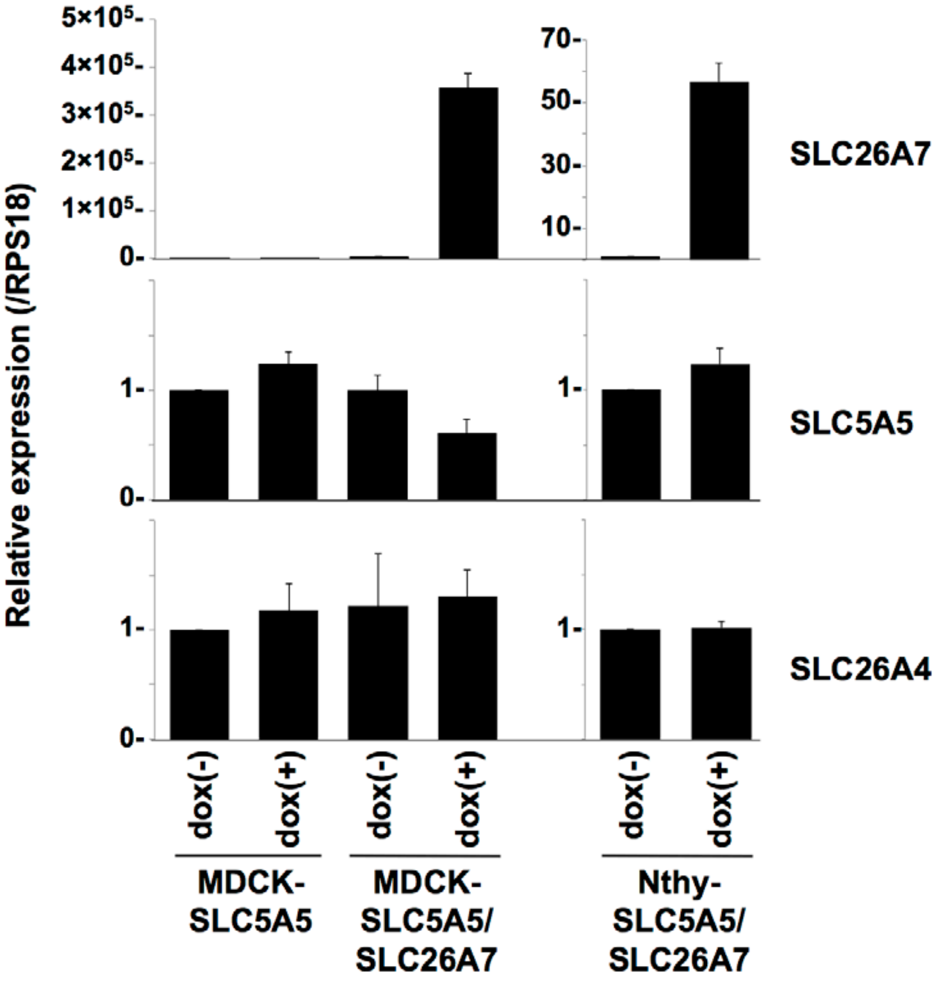

Supplementary Figure 5. Semiquantitative RT-PCR analysis of SLC26A7, SLC5A5, and SLC26A4 mRNA expression in SLC5A5-inducible or SLC5A5 and SLC26A7 (SLC5A5/SLC26A7)-inducible MDCK and Nthy cells. Data are shown as means±SD. dox, doxycycline. SLC5A5 and SLC26A4 expression were not greatly affected by *SLC26A7* induction.

Supplementary Figure 6

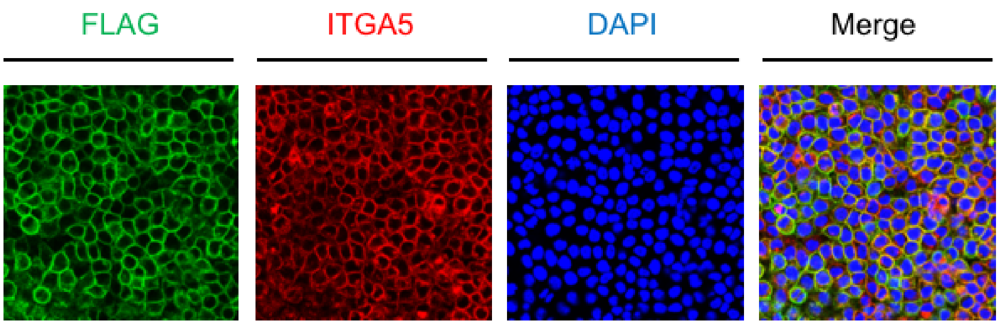

Supplementary Figure 6. Immunofluorescence analysis of FLAG (green) and ITGA5 (integrin subunit alpha 5, red) in FLAG-tagged SLC26A7 WT-transfected HeLa cells. 4′,6-diamidino-2-phenylindole, DAPI (blue) marks cell nuclei. Note SLC26A7 WT protein colocalized with the plasma membrane protein IGTA5.

Supplementary Figure 7

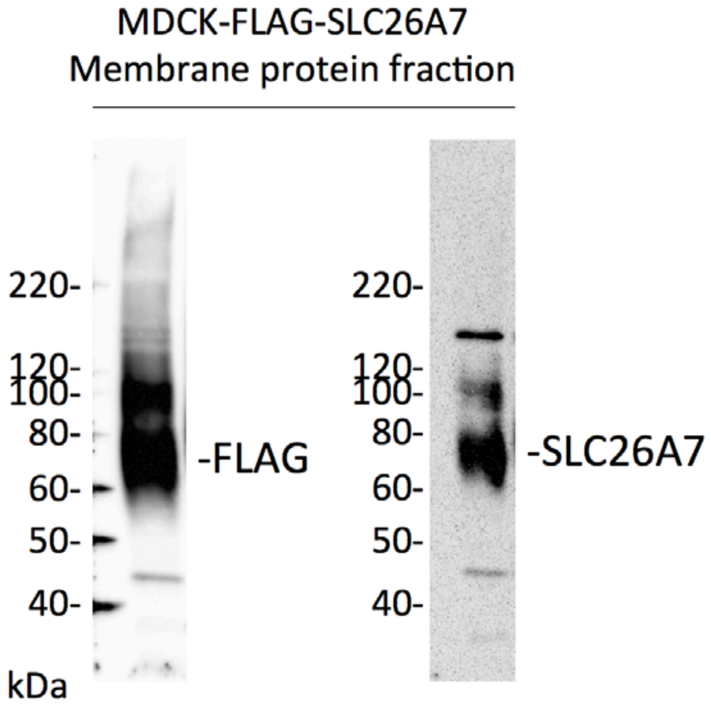

Supplementary Figure 7. Immunoblot analysis to confirm whether SLC26A7 is expressed on the cell membrane. Membrane protein fraction was extracted using a membrane protein isolation kit (Pierce Cell Surface Protein Isolation Kit, Thermo Fisher Scientific) from FLAG-tagged SLC26A7-inducible MDCK cells. FLAG-tagged SLC26A7 was detected in membrane protein fraction.

Supplementary Figure 8

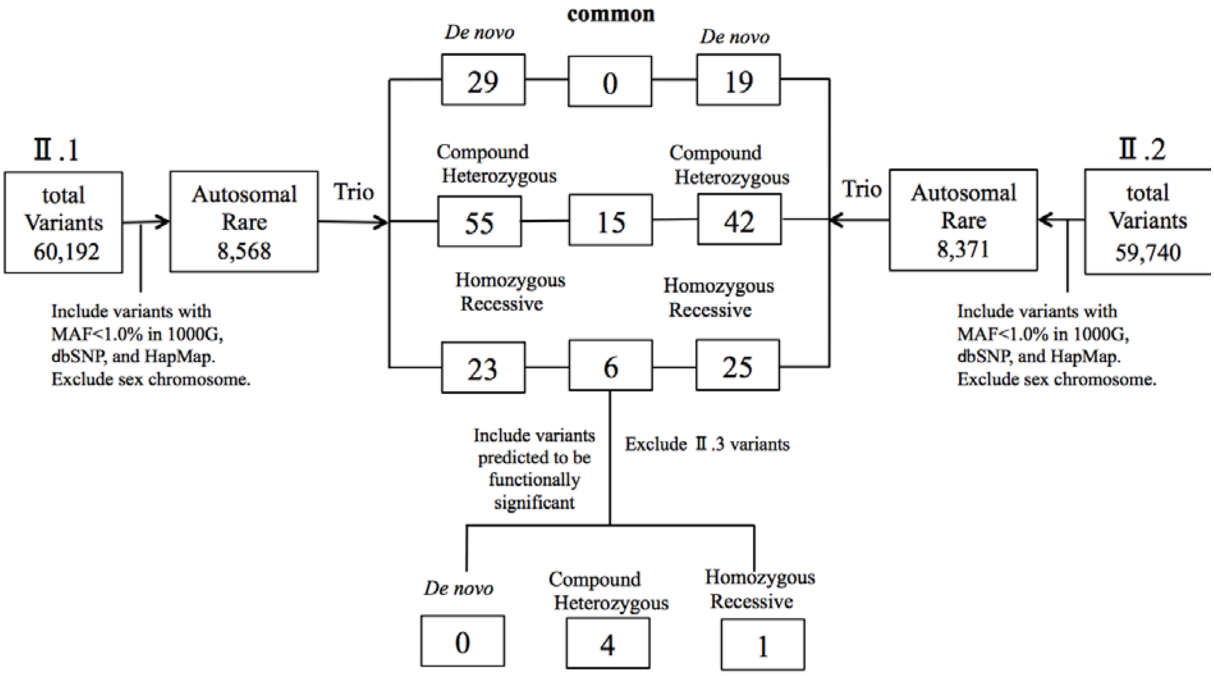

Supplementary Figure 8. Schematic for identifying candidate homozygous or compound heterozygous rare variants in genes.

Supplementary Figure 9

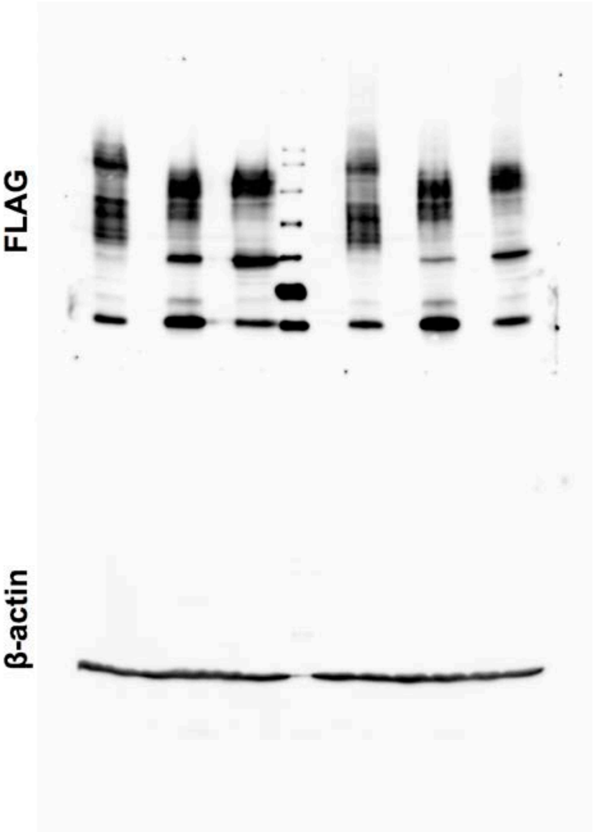

Supplementary Figure 9. Uncropped blot image in Figure 5a.

Supplementary Figure 10

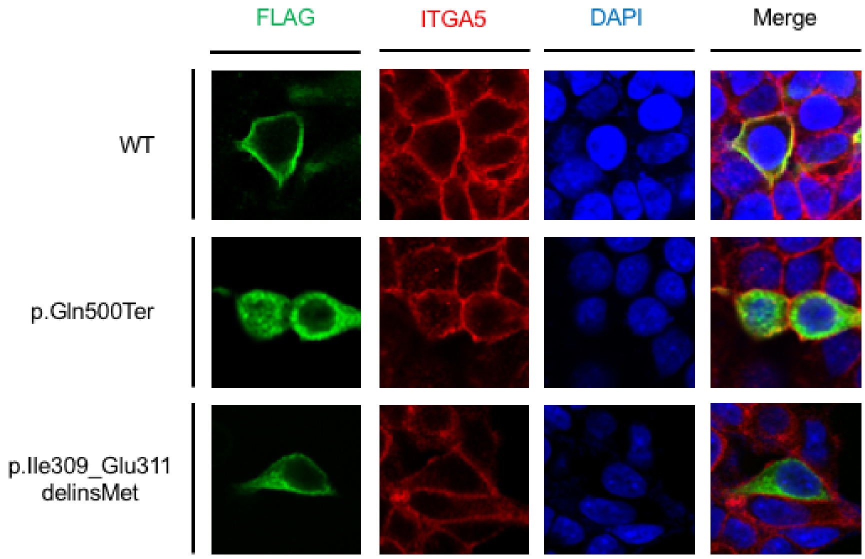

Supplementary figure 10. Immunofluorescence analysis of FLAG (green) and ITGA5 (red) in FLAG-tagged wild type (WT), p.Gln500Ter mutant or p.Ile309_Glu311delinsMet mutant SLC26A7 in transiently transfected HEK293T cells. 4′,6-diamidino-2-phenylindole, DAPI (blue) marks cell nuclei. WT protein colocalises with the plasma membrane protein IGTA5, however mutant proteins show predominantly cytosolic localisation.

Supplementary Figure 11

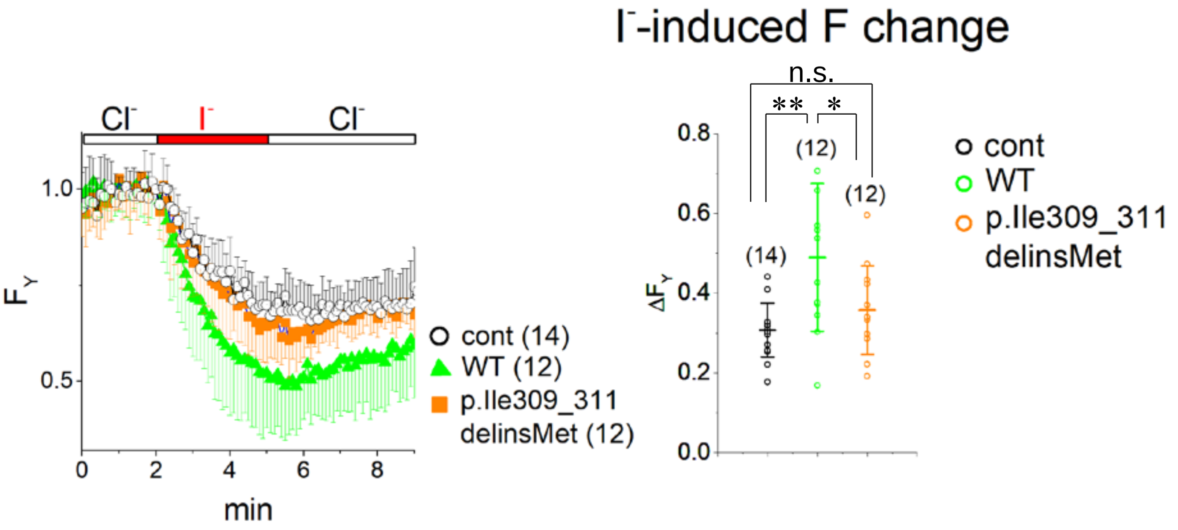

Supplementary Figure 11. (Left panel) The accumulation of I−. Shown are time-lapse changes in the normalised fluorescence intensity of YFP-HQ/IL (F_Y) in cells expressing the mutant YFP-HQ/IL alone (open circles) or YFP-HQ/IL with WT (green triangles) or p.Ile309_Glu311delinsMet mutant SLC26A7 (orange squares). Symbols and error bars represent mean and S.D., respectively The NaI bath solution was perfused for 3 minutes, as indicated by red bars. Numbers of cells are indicated in parentheses.(Right panel) Summary of the F_Y change. Circles represent changes in F_Y in each cell at 3 minutes after the NaI perfusion. Bars and error bars represent mean and S.D., respectively. Numbers of cells are indicated in parentheses. *, p<0.05; **, p<0.01; n.s., not significant (WT against control: *P*=0.0020, Tukey's test; WT against p.Il309_Glu311delinsMet mutant: *P*=0.0359, Tukey's test)

Supplementary Figure 12

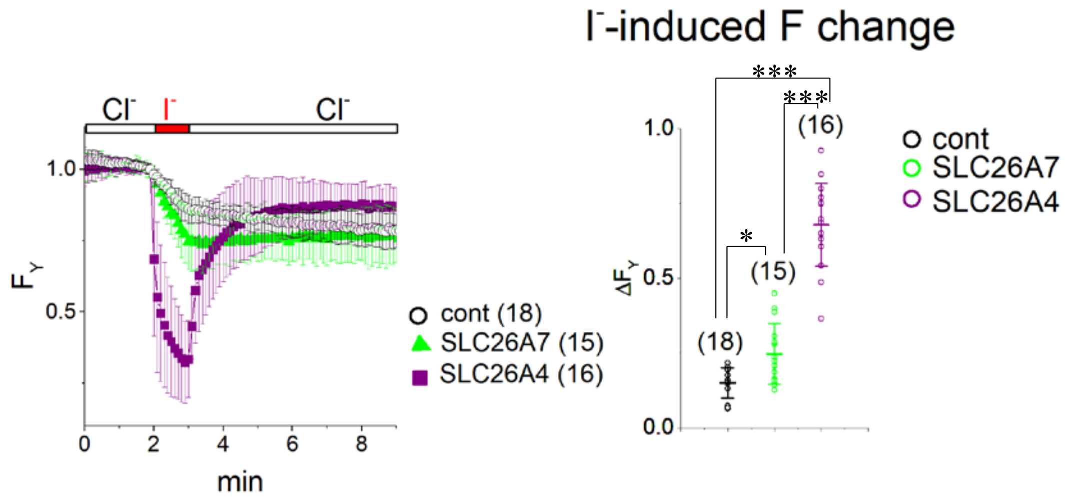

Supplementary Figure 12. (Left panel) The accumulation of I^- is compared in cells expressing the mutant YFP-HQ/IL alone or YFP-HQ/IL with SLC26A7 WT (green triangles) or SLC26A4 WT (violet squares). Symbols and error bars represent mean and S.D., respectively The NaI bath solution was perfused for 1 minute, as indicate by red bars. Numbers of cells are indicated in parentheses. (Right panel) Summary of the F_Y change. Circles represent changes in F_Y in each cell at 1 minute after the NaI perfusion. Bars and error bars represent mean and S.D., respectively. Numbers of cells are indicated in parentheses. *, p<0.05; ***, p<0.001 (Tukey’s test)

Supplementary Table 1

SLC26A7 expression in thyroid tissues. Abbreviations: +, positive; −, negative.

	SLC26A7 immunohistochemistry	
Tissue	Cytosol	Apical side
Thyroid-1	+	+
Thyroid-2	+	+
Thyroid-3	+	−
Thyroid-4	+	−
Thyroid-5	+	+
Thyroid-6	+	+
Thyroid-7	+	+
Thyroid-8	+	−
Thyroid-9	+	−
Thyroid-10	+	+

Tissue	SLC26A7 RT-PCR
Thyroid-4	+
Thyroid-5	+
Thyroid-7	+
Thyroid-10	+
Kidney-1	+
Kidney-2	+
Kidney-3	−
Kidney-4	−
Kidney-5	+
Kidney-6	+
Kidney-7	+

Supplementary Table 2

Cell line	Total iodine in cells (μg/L)
COS-7-SLC26A7 dox(-)	<25
COS-7-SLC26A7 dox(+)	<25
COS-7-SLC26A7 dox(-) SLC5A5 transfected	520
COS-7-SLC26A7 dox(+) SLC5A5 transfected	425
Nthy-SLC26A7 dox(-)	<25
Nthy-SLC26A7 dox(+)	<25
Nthy-SLC26A7 dox(-) SLC5A5 transfected	178
Nthy-SLC26A7 dox(+) SLC5A5 transfected	120

dox, doxycycline
